# Molecular and ultrastructural characterization of *Haplosporidium diporeiae* n. sp., a parasite of *Diporeia* sp. (Amphipoda, Gammaridea) in the Laurentian Great Lakes (USA)

**DOI:** 10.1186/1756-3305-7-343

**Published:** 2014-07-24

**Authors:** Andrew D Winters, Mohamed Faisal

**Affiliations:** Department of Fisheries and Wildlife, Michigan State University, 48824 East Lansing, Michigan USA; Pathobiology and Diagnostic Investigation, Michigan State University, 48824 East Lansing, Michigan USA

## Abstract

**Background:**

The phylum Haplosporidia contains coelozoic and histozoic, spore-forming, obligate protozoan endoparasites that infect a number of freshwater and marine invertebrates including bivalves, crustaceans, and polychaetes. In amphipods, haplosporidians cause systemic infection resulting in a range of pathologies. While amphipods belonging to the genus *Diporeia* (Gammarideae) have been shown to host haplosporidians, the taxonomic relationship of the *Diporeia* haplosporidian(s) is largely unknown due to the lack of phylogenetic and detailed ultrastructural studies.

**Methods:**

The infection characteristics and taxonomic identity of a haplosporidian infecting *Diporeia* spp. (*Diporeia*) were based on microscopical investigation, electron microscopy, and Bayesian phylogenetic inference using haplosporidian 16S rRNA gene sequences.

**Results:**

In stained sections, the haplosporidian was observed to cause systemic infections in *Diporeia* that were often accompanied with host tissue degeneration. The haplosporidian appeared as binucleate plasmodia and sporocysts containing different spore maturation stages in the coelom, connective tissue, digestive tissue, and muscle. All of the observed systemic infections progressed to sporogenesis. Transmission electron microscopy revealed that fixed mature spores were slightly ellipsoidal and had a mean spore length X width of 5.34 ± 0.17 × 4.09 ± 0.15 μm. A hinged opercular lid with a length of 3.1 ± 0.17 μm was observed for a number of developing spores. The average thickness of the cell wall was 90.0 ± 8.33 nm. Thin filaments (70 nm) composed of spore wall material were observed projecting from an abopercular thickening of the spore wall. Phylogenetic analysis showed that the haplosporidian is novel bearing some similarities with the oyster pathogen *Haplosporidium nelsoni*, yet distinctly different.

**Conclusions:**

Based on its morphology, genetic sequence, and host, it became evident that the *Diporeia* haplosporidian is taxonomically novel and we propose its nomenclature as *Haplosporidium diporeiae.* This is the first report of a haplosporidian infecting *Diporeia* in Lake Superior.

## Background

Haplosporidians are spore-producing, endoparasitic protists that parasitize a range of marine [1] and freshwater [2–4] invertebrates. Currently, four genera have been assigned to the phylum- *Urosporidium*, *Minchinia*, *Bonamia*, and *Haplosporidium*, and generic assignment has been largely determined by presence or absence of a hinged operculum on the outer surface of the anterior orifice and the origin of spore wall ornamentation [5–9]. *Urosporidium* are characterized by an internal flap of spore wall material covering the spore orifice, while the other three genera exhibit an external hinged operculum. *Minchinia* have cytoplasmic extensions originating from the epispore that disappear at the end of sporulation while *Bonamia* and *Haplosporidium* have ornamentation that is derived from the spore wall. Although recent molecular phylogenetic studies have confirmed the monophyly of Haplosporidia and shed light on the diversity of each genera, including *Bonamia* for which spores have only been characterized in one species [6, 10], they revealed an incongruence of molecular phylogeny of the Haplosporidia and the generic definition based on morphological characterization [7, 11].

In mollusks, haplosporidians have been reported to cause a range of lesions, from destruction of gills, gonads, or digestive gland to general destruction of all associated tissues [4, 12–15]. In the brackish environment, *Haplosporidium nelsoni*, the causative agent of MSX disease, has contributed to major mortalities in the eastern oyster (*Crassostrea virginica*) populations along the eastern coast of the United States for decades [16]. In the freshwater environment, *Haplosporidium pickfordi* was found infecting the digestive gland of snails in multiple lakes in northern Michigan, USA [17, 18] with limited evidence that the parasite is pathogenic to its hosts. Additionally, one species (*H. raabei*) was reported in the connective tissue of the gills, gonads, and digestive gland of zebra mussels (*Dreissena polymorpha*) from the Rhine and Meuse river basins in France, Germany, and the Netherlands [4].

Less is known regarding haplosporidian infections in amphipods. In *Rivulogammarus pulex*, *Haplosporidium gammari* has been shown to develop systemically in adipose tissue and destroy fat cells [19]. In *Parhyale hawaiensis*, infections by two different unidentified haplosporidians were associated with tissue damage that ranged from stretching, swelling, and fusion to vacuolation, fragmentation, and liquefaction necrosis of digestive epithelial cells and necrosis and rupture of skeletal muscle fibers surrounding the digestive canal and hepatopancreas [20]. Additionally, a range of microorganisms including unidentified haplosporidians have been observed infecting amphipods belonging to the genus *Diporeia*, a genus of amphipods for which taxonomic differences have not been resolved [[3, 21, 22] Nalepa and Faisal: Mechanistic approach to identify the role of pathogens in causing *Diporeia* decline in the Laurentian Great Lakes, Final Report, submitted]. The aim of the current study is to use microscopical and molecular techniques to determine the infection characteristics and phylogenetic relationship of a haplosporidian infecting *Diporeia* spp. (*Diporeia*) in the Laurentian Great Lakes (USA).

## Methods

### Sample collection and morphological examination

**Figure 1:**
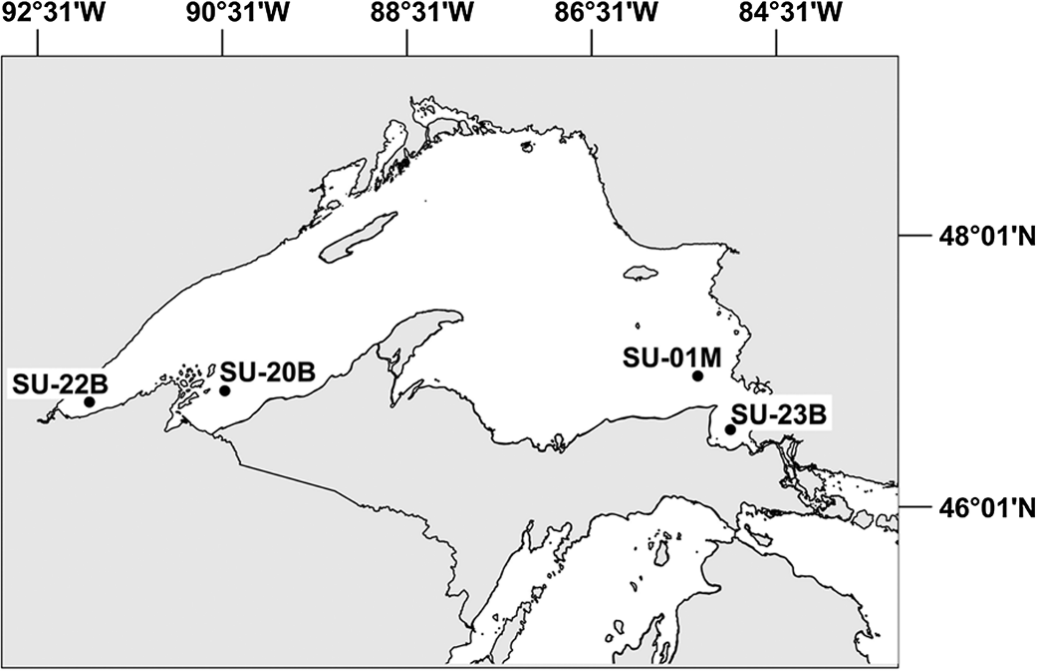
**Sampling sites in Lake Superior (USA) where** ***Diporeia*** **specimens were examined for a haplosporidian infection.**

In August 2008, a total of 332 *Diporeia* from four sites in Lake Superior (Figure 1) and a single pooled *Diporeia* sample (10 amphipods) from a site in Lake Michigan (45°10.7000 N, 086°22.5403 W) were collected for determining the presence of haplosporidian infection. Samples were collected by taking Ponar grabs (sampling area 9 × 9”/ 8.2 liters) at depths between 18-136 meters). Benthic samples were sieved (mesh = 0.25 mm) and *Diporeia* were identified according to [23] and placed in either 10% neutral buffered formalin for histopathological analysis or filter-sterile (0.2 μm) 80% ethanol for molecular analysis. Due to the low density of amphipods at the sites sampled and the low prevalence of haplosporidian infection in *Diporeia* [21], wet mounts of fresh samples were not prepared and screened for haplosporidian infection. Therefore, fresh haplosporidians were not immediately preserved in a glutaraldehyde solution for ultrastructural studies using transmission electron microscopy.

For histopathological analysis, amphipods preserved in formalin were dehydrated in a graded series of alcohols, embedded in paraffin, cut into 3-4 μm thick serial sections, and stained with hematoxylin and eosin. An average of 83 amphipods was sampled from each site. The taxonomic system for haplosporidia infecting *Diporeia* was based on the morphological criteria used for taxonomy detailed in [24]. To ascertain morphological similarities of haplosporidians infecting Great Lakes *Diporeia*, we compared the haplosporidian development stages observed in Lake Superior *Diporeia* with 1) those observed in samples of *Diporeia* collected from nine sites in southern Lake Michigan between 1980 and 2007 (Nalepa and Faisal: Mechanistic approach to identify the role of pathogens in causing *Diporeia* spp. decline in the Laurentian Great Lakes, Final Report, submitted) and 2) those reported in [3, 21].

Ultrastructural studies were performed on representative, infected *Diporeia* samples collected from site SU-23B in Lake Superior that were embedded in a paraffin block. Samples were deparaffinized, post-fixed, and processed for transmission electron microscopy (TEM). For TEM, ultra-thin sections (60–100 nm) were stained with 2% (w/v) uranyl acetate in 50% ethanol followed by Reynold’s lead citrate and examined in a JEM-100 CX II electron microscope at an accelerating voltage of 100 kV.

### DNA isolation, amplification, and sequencing

Genomic DNA from an infected *Diporeia* collected from the same sampling station in Lake Superior was extracted using the DNeasy DNA extraction kit (QIAGEN) according to the manufacturer’s instructions. PCR amplification of 16S rDNA was amplified according to the protocol of [4]. Specifically, the HAP-F1 + 16S-B primer set was initially used to screen Lake Superior *Diporeia* populations for haplosporidian 16S rRNA genes. A negative control containing no DNA was included in the PCR reaction. The resulting PCR products were visualized by agarose gel electrophoresis to confirm only a single fragment was amplified, cloned using a TOPO TA Cloning Kit® (Invitrogen, CA, USA) following the manufacturer’s protocol, cultured on Luria-Bertani agar plates (Fisher Scientific Inc., PA, USA) containing 50 μg/ml Kanamycin as directed by the manufacturer’s protocol, and sequenced using the M13f (5′-GTT TTC CCA GTC ACG AC-3′), M13r (5′-CAG GAA ACA GCT ATG ACC-3′) and amplification primers. The resulting sequences were deposited in GenBank (Accession numbers: KF378734 and KM058119.

### Sequence and phylogenetic analyses

The 16S rRNA gene sequences were submitted for BLAST (National Center for Biotechnology Information) searches and highly similar matches were included in the dataset for phylogenetic analysis. Selection of sequences to include in phylogenetic analyses was based on the findings of [9, 25]. A total of 19 haplosporidian and three non-haplosporidian outgroup 16S rRNA gene sequences were aligned with ClustalW as implemented in MEGA 5.0 [26] using default settings. The average length of sequences in the final alignment file was 1,754 bp. The alignment file was visually checked for alignment gaps and missing data in nucleotide positions.

Prior to phylogenetic analysis, the program jModelTest [27] was used to select the best fitting substitution model according to the corrected Akaike information criterion (AICc) [28]; the model with the lowest AICc value was identified as the best model in terms of fit and parsimony [29]. A total of 1,624 candidate models, including models with equal/unequal base frequencies, with/without a proportion of invariable sites (+I), and with/without rate variation among sites (+G) were tested. The best-fit model of nucleotide substitution was the transitional model [30] with γ distributed rates (TIM + I + G) with unequal base frequencies.

Tree topologies were inferred using a Bayesian approach using MRBAYES v 3.1.2 [31]. For Bayesian analysis, we used the GTR + I + G model of nucleotide substitution, given that it is the model available in MRBAYES that best matches the TIM + I + G model. Bayesian analysis included four Monte Carlo Markov chains (MCMC) for 2,000,000 generations, and trees sampled every 1,000th generation. The first 25% of samples were discarded as burn-in. After discarding the burn-in samples, the remaining data were used to generate a 50% majority-consensus tree.

## Results

### Pathology and morphological characterization

**Figure 2:**
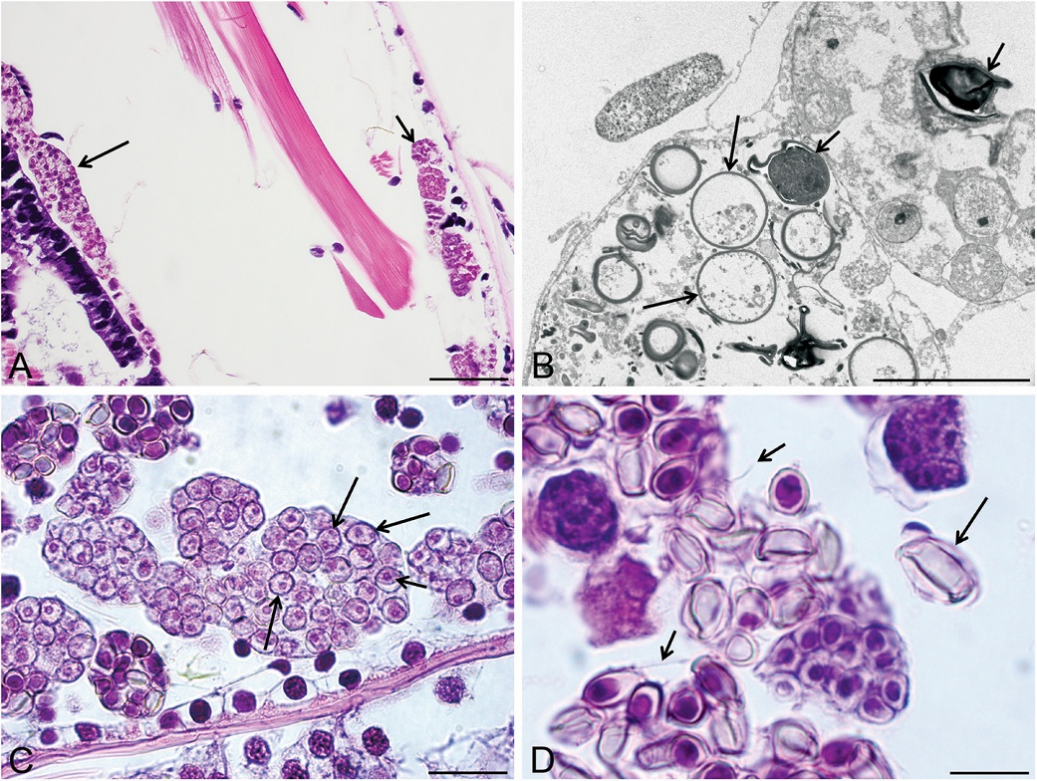
**Histological sections of haplosporidian in** ***Diporeia*** **collected from Lake Superior (USA). (A)** Plasmodia undergoing various stages of shizogeny lining the epicuticle (small arrow) and sporocysts containing developing spores lining host digestive tissue (large arrow). **(B)** Transmission electron micrograph of sporocysts containing both amorphous, immature spores (small arrows) and mature spores (large arrows). **(C)** Sporoblast containing two basophilic bodies (large arrows). Note the sporoblast with a well-defined endosporoplasm (small arrow). **(D)** Mature spore that has been liberated from the sporocyst (large arrow) and thin filaments projecting from the end of multiple developing spores (small arrows). Scale bars: A = 75 μm, B = 10 μm, C = 25 μm, and D = 5 μm.

Haplosporidian infections were observed in *Diporeia* collected from all four sites in Lake Superior (Table 1). Prevalence ranged from 1.08% in site SU-22B, to 2.97% in site SU-20B making an overall prevalence of 2.11%. In all infected *Diporeia* (7/7), haplosporidian developmental stages were widespread and distributed in a systemic way and all had progressed to sporogenesis. Plasmodial development and sporogenesis was relatively synchronous. For all observed infections, plasmodia and developing spores were observed in high densities throughout the coelom. Additionally, developing spores were often associated with connective, digestive, and muscle tissues. Plasmodia and sporocysts were commonly observed lining the epicuticle and digestive tissue (Figure 2A). Infections were often accompanied by degeneration of host tissues. An apparent host response to infection was observed as differentiated, melanized circulating host hemocytes surrounding sporocysts.

**Table 1:** **Samples in which haplosporidian infection was observed in sections of** ***Diporeia*** **collected from Lake Superior in 2008**

Site	Coordinates	Depth (m)	Month/year sampled	Prevalence
SU-01 M	46.99°N & 85.16°W	95	8/2008	2.94% (2/68)
SU-20B	46.88°N & 90.28°W	113	8/2008	2.97% (3/101)
SU-22B	46.80°N & 91.75°W	53	8/2008	1.08% (1/93)
SU-23B	46.60°N & 84.81°W	60	8/2008	1.43% (1/70)

Stained, paraffin-embedded sections revealed that the haplosporidian spores within Lake Superior *Diporeia* were contained in round to amorphous sporocysts averaging 29.09 ± 3.20 μm (n = 8) in length ranging from 19.94 to 42.47 μm. TEM of the sample collected from SU-23B revealed the presence of both immature spores that were amorphous and electron-dense and spherical to slightly ellipsoidal mature spores that exhibited a lower electron density within sporocysts (Figure 2B).

**Figure 3:**
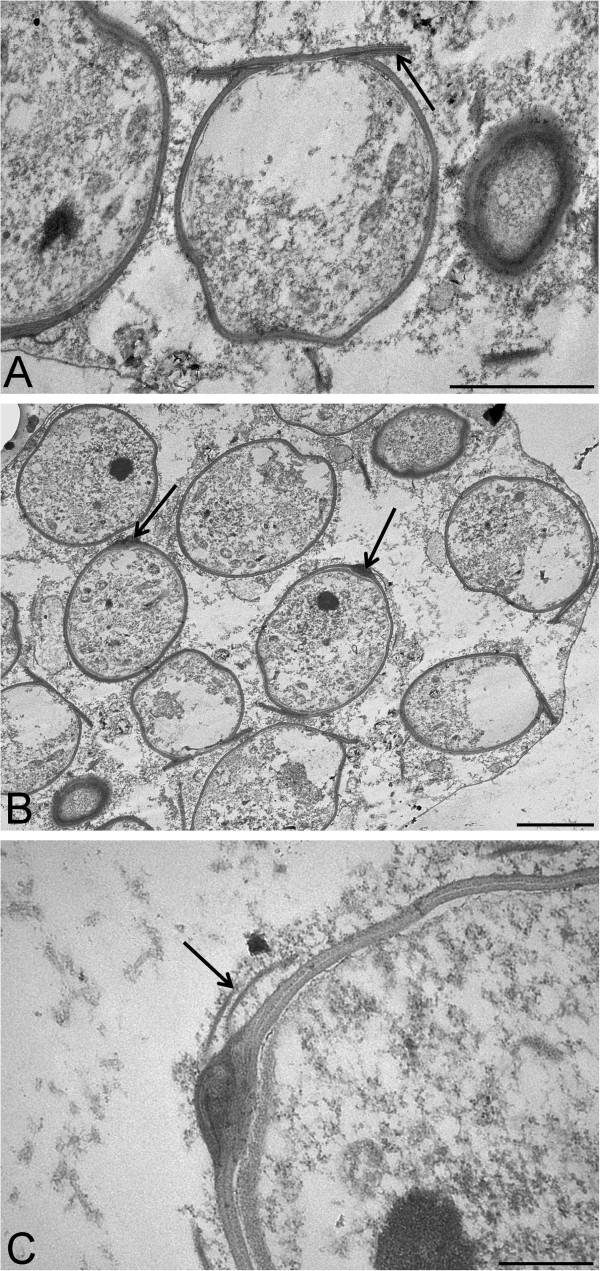
**Transmission electron micrographs of haplosporidian infecting** ***Diporeia*** **in Lake Superior. (A)** Spore with a hinged opercular lid (arrow). **(B)** Spores exhibiting a thickening of the spore wall at the abopercular end (arrows). **(C)** Mature spore exhibiting what appears to be thin filaments projecting from a thickening of the cell wall (arrow). Scale bars: A = 2,000 nm, B = 2,500 nm, and C = 500 nm.

In stained histological sections, sporoblasts with a well-defined endosporoplasm often containing two basophilic bodies were observed (Figure 2C). Sporocysts often contained mature spores with a well-defined, basophilic endosporoplasm. As described for *Haplosporidium* by [32], mature spores were observed outside of sporocysts and multiple spores showed what appeared to be thin filaments projecting from the abopercular end of the spore (Figure 2D).By TEM, individual spores measured 5.34 ± 0.17 μm long by X 4.09 ± 0.15, μm wide (n = 14). The average thickness of the cell wall was 90.0 nm (SE = 8.33, n = 6). A hinged opercular lid with a length of 3.1 ± 0.17 μm (n = 6) composed of spore wall material was observed for a number of developing spores (Figure 3A). A thickening of the spore wall at the abopercular end was observed for several mature spores (Figure 3B). Thin filaments (70 nm) that appear to be composed of spore wall material appeared to project from the abopercular thickening (Figure 3C).

### Phylogenetic analysis

**Figure 4:**
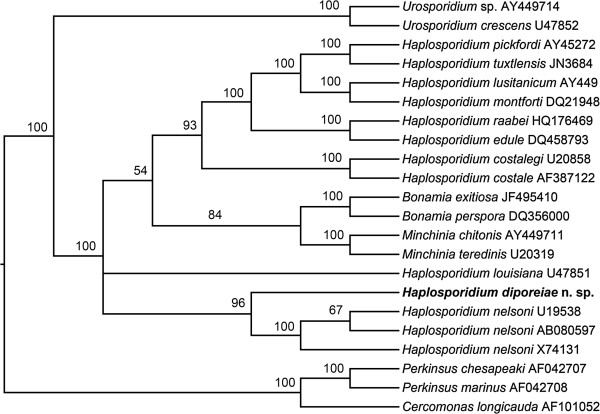
**Phylogenetic tree (50% majority-rule consensus) based on Bayesian Inference (MrBayes 3.1.2) of Haplosporidia based on the small subunit ribosomal gene.** Numbers at the nodes are Bayesian posterior probabilities. *Cercomonas longicauda*, *Perkinsus chesapeaki*, and *P. marinus* were used as an outgroup for Haplosporidia based on the results of Reece *et al.*[9].

The 16S rDNA sequences obtained from haplosporidians infecting multiple *Diporeia* in Lakes Superior (1,522 bp) and Michigan (545 bp) were identical over the nucleotide positions covered by both sequences. Pairwise comparison of the consensus 16S rDNA sequence of the haplosporidian obtained from *Diporeia* to other closely related haplosporidians showed that the *Diporeia* haplosporidian was most similar to *Haplosporidium nelsoni* sequences (GenBank Accessions U19538 and AB080597). The resulting tree of phylogenetic inference had the genus *Haplosporidium* as paraphyletic as previously reported [9, 25], and showed that the sequence obtained from the *Diporeia* haplosporidian aligned with *H. nelsoni* but was distinctly different (Figure 4). Posterior probabilities of branching points based on Bayesian inference indicated that the node support of the Lake Superior *Diporeia* taxon was 96%, strongly suggesting that the haplosporidian infecting Lake Superior *Diporeia* is a novel species of *Haplosporidium*.

## Discussion

Results of both morphological (Table 2) and molecular (Table 3) analyses indicate the *Diporeia* haplosporidian is novel. In comparison to the *Diporeia* haplosporidian, all similar haplosporidian strains had a 16S rDNA sequence dissimilarity of 1.2% or greater indicating the *Diporeia* haplosporidian is novel. While phylogenetic analysis showed that the *Diporeia* haplosporidian was most similar to the oyster pathogen *H. nelsoni*, the *Diporeia* haplosporidian formed a separate clade in the tree with high node support (96% posterior probability) suggesting it is distinctly different from *H. nelsoni*. The phylogenetic relationship between these two haplosporidians is also reflected in their spore morphologies as well since both have similar spore ornamentation (i.e., filaments composed of spore wall material projecting away from the spore wall) however, the spores of the haplosporidian observed infecting *Diporeia* are smaller than those of *H. nelsoni*.

**Table 2:** **Morphological characteristics of all described freshwater species of Haplosporidia and those of** ***Haplosporidium nelsoni*** **compared to** ***H. diporeiae*** **n. sp**

***Haplosporidium***sp.	Host	Mean spore length, range (μm)	Mean spore width, range (μm)	Location	Reference
*H. diporeiae* n. sp.*	*Diporeia* spp.	5.3	4.1	Lake Superior (USA)	This study
		4.4-6.7	3.0-4.9		
*Haplosporidium* sp.*	*Diporeia* spp.	8.1 range not stated	6.1 range not stated	Lakes Michigan and Huron	Messick et al. [21]
*Haplosporidium* sp.*	*Diporeia* spp.	8.1 ± SE 0.23 range not stated	6.1 ± SE 0.16 range not stated	Lakes Michigan and Huron	Messick, [3]
*H. nelsoni*	Oysters *Crassostrea virginica*, *C. gigas*	8.1 5.3-10.7	5.5 4.8-7.5	East Coast of North America	Perkins, [33]
*H. cernosvitovi*	Oligochaetes *Opistocysta flagellum*	Mean not stated 10–11	Mean not stated 6–7		Jírovic, [34]
*H. limnodrili*	Oligochaetes *Limnodrilus udekemianu*	Mean not stated 10–12	Mean not stated 8–10		Granata, [35]
*H. pickfordi*	Snails *Physella parkeri*, *Lymnaea stagnalis*, and *Heliosoma companulatum*	8 · 9	4 · 5	Lakes in northern Michigan, USA	Barrow, [17]
		8 · 5–10 · 4	3 · 8–4 · 6		
*H. raabei*	Zebra mussels *Dreissena polymorpha*	7 · 5	5 · 2	Meuse River (France)	Molloy *et al*. [4]
		6 · 5–9 · 4	4 · 5–5 · 9		
*H. vejdovskii*	Oligochaetes *Mesenchytraeus flaviduus*	Mean not stated 10–12	Mean not stated 8–10		Caullery and Mesnil, [36]

(*) indicates haplosporidians for which spore measurements were made from fixed material.

**Table 3:** **Pairwise genetic distances between** ***Haplosporidium diporeiae*** **n. sp. and similar haplosporidians based the partial 16S small subunit rDNA sequences used for phylogenetic analysis**

*Haplosporidium diporeia* n. sp.																					
*H. nelsoni* (U19538)	0.12																				
*H. nelsoni* (AB080597)	0.13	0.00																			
*H. nelsoni* (X74131)	0.13	0.01	0.01																		
*H. costale* (AF387122)	0.13	0.15	0.15	0.15																	
*H. costalegi* (U20858)	0.14	0.15	0.15	0.15	0.01																
*H. edule* (DQ458793)	0.16	0.18	0.18	0.18	0.14	0.15															
*H. raabei* (HQ176469)	0.17	0.18	0.18	0.18	0.15	0.16	0.05														
*H. tuxtlensis* (JN368430)	0.18	0.19	0.19	0.19	0.16	0.17	0.17	0.17													
*H. lusitanicum* (AY449713)	0.17	0.17	0.17	0.17	0.15	0.15	0.17	0.17	0.09												
*H. montforti* (DQ219484)	0.17	0.17	0.17	0.17	0.15	0.15	0.17	0.17	0.09	0.00											
*H. pickfordi* (AY452724)	0.18	0.18	0.18	0.18	0.15	0.16	0.15	0.15	0.04	0.09	0.09										
*H. louisiana* (U47851)	0.25	0.25	0.25	0.25	0.25	0.25	0.26	0.26	0.27	0.26	0.26	0.27									
*Bonamia exitiosa* (JF495410)	0.13	0.14	0.14	0.14	0.14	0.14	0.15	0.16	0.17	0.15	0.15	0.16	0.25								
*Bonamia perspora* (DQ356000)	0.14	0.15	0.15	0.15	0.14	0.15	0.16	0.16	0.18	0.16	0.16	0.16	0.25	0.03							
*Minchinia chitonis* (AY449711)	0.16	0.17	0.17	0.17	0.17	0.17	0.19	0.18	0.20	0.19	0.19	0.20	0.26	0.16	0.17						
*Minchinia teredinis* (U20319)	0.18	0.17	0.17	0.17	0.17	0.17	0.18	0.18	0.18	0.18	0.18	0.17	0.26	0.16	0.16	0.11					
*Urosporidium* sp. (AY449714)	0.20	0.21	0.21	0.21	0.21	0.21	0.22	0.22	0.24	0.22	0.22	0.23	0.27	0.21	0.21	0.22	0.24				
*Urosporidium crescens* (U47852)	0.20	0.21	0.21	0.21	0.21	0.22	0.22	0.22	0.24	0.22	0.22	0.23	0.28	0.21	0.22	0.22	0.23	0.04			
*Perkinsus chesapeaki* (AF042707)	0.22	0.23	0.24	0.24	0.23	0.23	0.24	0.26	0.24	0.23	0.23	0.24	0.30	0.23	0.24	0.23	0.25	0.22	0.22		
*Perkinsus marinus* (AF042708)	0.22	0.24	0.24	0.24	0.23	0.23	0.25	0.26	0.25	0.24	0.24	0.24	0.30	0.23	0.24	0.23	0.25	0.23	0.22	0.02	
*Cercomonas longicauda* (AF101052)	0.24	0.24	0.24	0.24	0.24	0.24	0.25	0.25	0.26	0.24	0.24	0.25	0.30	0.22	0.23	0.24	0.24	0.22	0.22	0.17	0.17

As with *H. nelsoni*, the *Diporeia* haplosporidian can be distinguished from the other five recognized freshwater species by its smaller spore size (Table 2). In comparison to *H. pickforii*, both haplosporidians are reported to infect invertebrates in Michigan (USA) and have an abopercular thickening of the spore wall [2]. However, in addition to having somewhat smaller spores, the *Diporeia* haplosporidian has thinner filaments than that reported for *H. pickfordi* [2]. Additionally, phylogenetic analysis shows that the two species are considerably different (82% sequence similarity) (Table 3).

The finding that identical 16S rDNA sequences were obtained from haplosporidians infecting multiple *Diporeia* in Lakes Superior and Michigan suggests that the same haplosporidian infects *Diporeia* populations residing within both the lakes. Additionally, the haplosporidian described in this study is morphologically similar to those previously described in Lakes Michigan and Huron *Diporeia* (i.e. spores with a hinged operculum and filaments emanating from a small thickening on the abopercular end) [3, 21] and thus may be the same species. Similarly, the observed prevalence of haplosporidian infections in Lake Superior *Diporeia* populations (1.1-3.0%) is relatively similar to those previously reported in *Diporeia* populations in Lake Michigan (0.3-2.0%) and Huron (1.0%) [21]. However, the haplosporidian infecting *Diporeia* from Lakes Michigan and Huron were only detected in the hemal sinuses with no obvious tissue damage or host response, whereas the haplosporidian infections observed in this study were detected in multiple tissues and were associated with degeneration of multiple tissues and a pronounced host response. Further investigation of the relatedness of haplosporidians infecting *Diporeia* in Lakes Superior and Michigan as well as elsewhere in the Laurentian Great Lakes Basin is warranted.

This is the first report of a haplosporidian infecting *Diporeia* in Lake Superior. Based on its morphology, genetic sequence, and host, this is a novel *Haplosporidium* sp., for which the name *Haplosporidium diporeiae* n. sp. is proposed.

## Conclusion

### Description of *Haplosporidium diporeiae*

#### Taxonomic summary

Phylum Haplosporidia [37]

Class Haplosporea

Order Haplosporida

Family Haplosporidiidae

*Haplosporidium diporeiae* n. sp.

### Description

Spherical to slightly ellipsoidal (5.34 ± 0.17 × 4.09 ± 0.15 μm) spores are present throughout coelom, connective, digestive, and muscle tissues. Binucleate plasmodia. Plasmodia contain two nuclei. Plasmodia and sporocysts are typically up to about 30 μm in width. Developing spores have a hinged opercular lid with a length of 3.1 ± 0.17 μm composed of spore wall material. The spore wall is thickened at the abopercular end with individual string-like projections.

### Type host

#### *Diporeia*sp.

Amphipoda, Gammaridae, Phoxocephaloidae, Haustoriidae

### Type locality

Lake Superior (USA)

Sampling site SU-23B (46.60°N & 84.81°W)

Depth = 60 m

### Type material

Reference materials are deposited at

The U.S. National Parasite Collection, U.S. Department of Agriculture, Beltsville, MD.

USNPC number 107252.00.

Ribosomal DNA sequence: Deposited to GenBank.

Accession number KF378734.

In accordance with section 8.5 of the ICZN’s International Code of Zoological Nomenclature, details of the new species have been submitted to ZooBank with the life science identifier (LSID) zoobank.org:pub:0B34CB35-B447-48AA-A0DA-C00E81EA7940.

### Etymology

The specific epithet refers the genus of the host *Diporeia*.
